# Application of the Milan System for Reporting Salivary Gland Lesions and Its Cytohistological Correlation for Risk Stratification: A Single-Institution Experience

**DOI:** 10.7759/cureus.47383

**Published:** 2023-10-20

**Authors:** Madhu Kumar, Pooja Dwivedi, Malti Kumari Maurya, Shalini Bhalla, Manish Chandra, Suresh Babu

**Affiliations:** 1 Pathology Department, King George's Medical University, Lucknow, IND; 2 Pathology Department, Sarojini Naidu Medical College, Agra, IND; 3 Otorhinolaryngology Department, King George's Medical University, Lucknow, IND

**Keywords:** fine needle aspiration cytology (fnac), cytohistological correlation, cell block, salivary gland cytology, milan system

## Abstract

Introduction: Salivary gland tumors are known to have a heterogeneous profile with variable clinical presentation and a wide variety of histological subgroups of prognostic significance. Immunocytochemical markers that aid in the diagnosis and characterization of the cell type of origin are critical for this heterogeneous group of malignancies.

Aims and objectives: To study the application of The ‘Milan System’ for Reporting Salivary Gland Cytology and the diagnostic utility of a panel of immunocytochemical markers in the diagnosis of salivary gland neoplasms and their cytohistological correlation for their risk stratification.

Materials and methods: This was a prospective study carried out in which a total of 60 patients were enrolled in the study. Fine-needle aspiration cytology (FNAC) smears and cell blocks were prepared with standard techniques and staining procedures. Immunocytochemistry (ICC) was performed on cell block sections by immunoperoxidase procedure. Immunocytochemical (ICC) stains were used for the differentiation of the lesions in cell blocks. Histopathology was also studied if the patient underwent excision of salivary gland lesions.

Discussion and results: Almost 60 cases were studied under FNAC and cell block evaluation, as well as ICC, among those five (8.33%) samples were inadequate, eight (13.3%) were non-neoplastic, 27 (45%) were benign, one (1.7%) was neoplasm with uncertain malignancy potential, one (1.7%) was suspected of malignancy, and 19 (31.7%) were malignant. The histopathological diagnosis was confirmed in 47 cases. Of these, 24 (51.1%) were benign and 23 (48.9%) were malignant. The malignancy rate for Milan Categories I, II, III, IVa, IVb, V, and VI was 0%, 0%, 100%, 24%, 50%, 80%, and 84.6%, respectively. The study showed that malignancy risk stratification could be further improved by using cell block with immunocytochemistry as a complementary diagnostic modality.

Conclusions: The present study was carried out to assess the usefulness of the Milan system to report salivary gland cytology results. Thus, the findings of the present study show that the Milan system is helpful in stratifying the risk of malignancy in salivary gland tumors.

## Introduction

Salivary gland fine-needle aspiration cytology (FNAC) is used worldwide to diagnose and manage salivary gland malignancies. It contains critical information about the tumor's origin (salivary/non-salivary), nature (benign/malignant), and severity (low grade/high grade). It is a minimally invasive, safe, cost-effective, and precise approach that is particularly valuable in classifying a large proportion of salivary gland nodules as benign, avoiding unnecessary surgical procedures in patients with benign diseases. In addition, it serves as a roadmap for future management strategies [1,2].

The American Society of Cytopathology and the International Academy of Cytology have proposed the "Milan System for Reporting Salivary Gland Cytopathology" (MSRSGC). It was introduced in 2018 and is a classification approach based on evidence to resolve these difficulties in salivary gland cytology. It calculates the risk of malignancy (ROM) for six diagnostic categories and makes clinical care recommendations [3]. Immunocytochemical markers that aid in the diagnosis and characterization of the cell type of origin are critical for this heterogeneous group of malignancies. In this study, we stratified all salivary gland lesions according to MSRSGC and thus tried to evaluate their diagnostic precision by cytohistological correlation and calculate the ROM for each category. The aim of the study is the application of the ‘Milan System’ for reporting salivary gland cytology and the expression of immunocytochemical markers in diagnosing salivary gland neoplasms and its cytohistological correlation for its risk stratification and to evaluate the precision and risk of diagnostic malignancy of various diagnostic categories.

## Materials and methods

This study was prospective conducted over a period of one year in the Department of Pathology in collaboration with the Department of Otorhinolaryngology. A total of 60 patients were included in the study who visited the cytology unit of the Department of Pathology for FNAC of salivary gland lesions. After recording the relevant clinical and radiological details, FNAC was performed and smears were stained with May-Grunwald-Giemsa and hematoxylin and eosin stains, and cell blocks were also prepared.

Cell blocks were prepared by adding material acquired from salivary gland lesion fine-needle aspiration cytology to 9 ml 100% alcohol, 1 ml glacial acetic acid, and 1 ml formalin. Following that, the mixture was centrifuged for 10 minutes at a speed of 2,000 rpm. For a period of 12 hours, the test tube was left unattended. Then the cell button was taken from the test tube, wrapped in filter paper and gauzed, then embedded in histokinette, and processed. Sections cut from these blocks and slides were stained with hematoxylin and eosin.

Immunocytochemical (ICC) markers were performed on cell block sections by immunoperoxidase procedure. A wide panel of ICC markers was carried out for exact subtyping if needed. Immunocytochemical markers were applied to those cases with adequate material present on the cell block. Pleomorphic adenoma gene 1 (PLAG1) is a zinc finger transcription factor and a proto-oncogene located on chromosome 8q12. PLAG1 fusion appears to be highly specific for pleomorphic adenoma (PA) and carcinoma (CA) ex-PA as it has not been detected in other benign or malignant salivary gland neoplasms. SOX-10 ICC may be helpful for diagnosing salivary gland neoplasms, which show acinus and intercalated duct differentiation. SOX-10 is a potential marker for acinar and intercalated duct differentiation in salivary gland tumors.

The rate of malignancy, sensitivity, specificity, positive predictive value (PPV), negative predictive value (NPV), and diagnostic precision were evaluated. The risk of malignancy was calculated by dividing the cases that turned out to be malignant on histopathology in each category by the total number of cases in each category in cytology.

Statistical analysis was performed using SPSS software (SPSS Inc., Chicago, IL, USA) for the Windows program (21.0 version). Dichotomous variables that were presented in number were analyzed using the Chi-square test. A p-value of < 0.05 or 0.001 was considered significant. All FNAC smears and their corresponding cell block sections were examined independently by two cytopathologists. The ethical clearance was sanctioned by the Institutional Ethics Committee of King George's Medical University, UP, India, and the reference code is 101st ECM IIB/64 via letter number 398/Ethics/2020.

## Results

A total of 60 patients with salivary gland lesions who met the inclusion criteria were enrolled in the study. The age of the patients ranged between 15 and 75 years, the mean age was 42.93±14.68 years, and the majority were aged 19-45 years (51.7%). The most common site of FNAC was the parotid (n=39; 65%) followed by the submandibular region/gland (n=14; 23.3%). The FNAC from palatal regions was n=3 (5%) patients, and FNAC from other sites was n=4 (6.7%) patients. The difference in age and gender ratio and laterality of patients in different Milan system categories was not found to be statistically significant.

**Figure 1:**
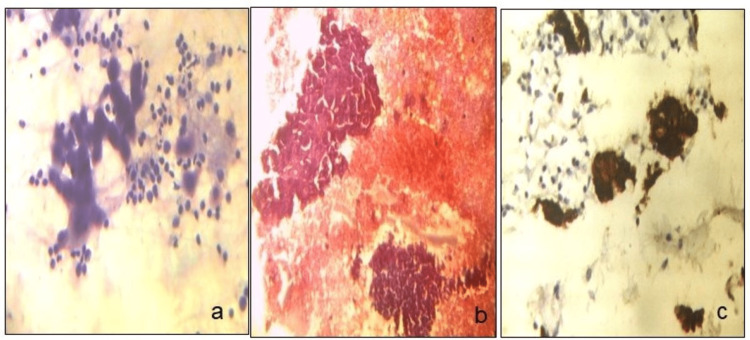
Stained cells. FNAC smears and cell block smears of Warthin’s tumor (a, b) (MGG stain, ×10 and H&E stain, ×20), and CK7 positive in epithelial cells on cell block (c) (ICC stain, ×20). FNAC: fine-needle aspiration cytology, MGG: May-Grunwald-Giemsa, H&E: hematoxylin and eosin, ICC: immunocytochemistry.

**Figure 2:**
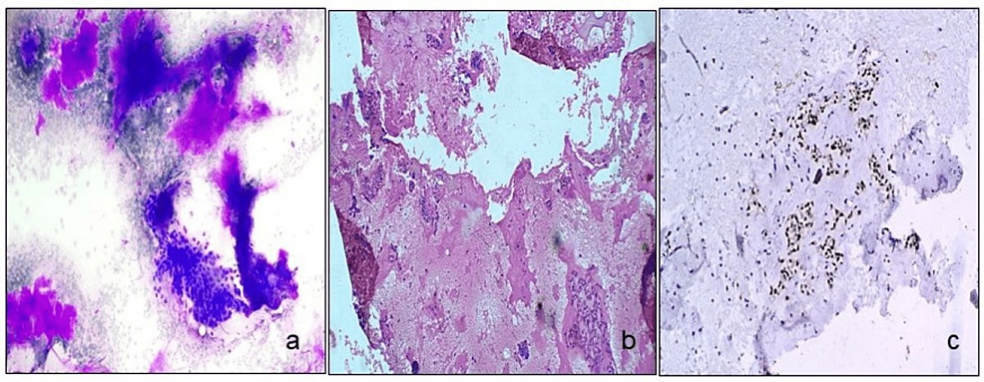
Stained cells. FNAC smears and cell block smears of pleomorphic adenoma (a, b) (MGG stain, ×10 and H&E stain,×20), and PLAG-1 positive in tumor cells on cell block (c) (ICC stain, ×20). FNAC: fine-needle aspiration cytology, PLAG-1: pleomorphic adenoma gene 1, MGG: May-Grunwald-Giemsa, H&E: hematoxylin and eosin, ICC: immunocytochemistry.

**Figure 3:**
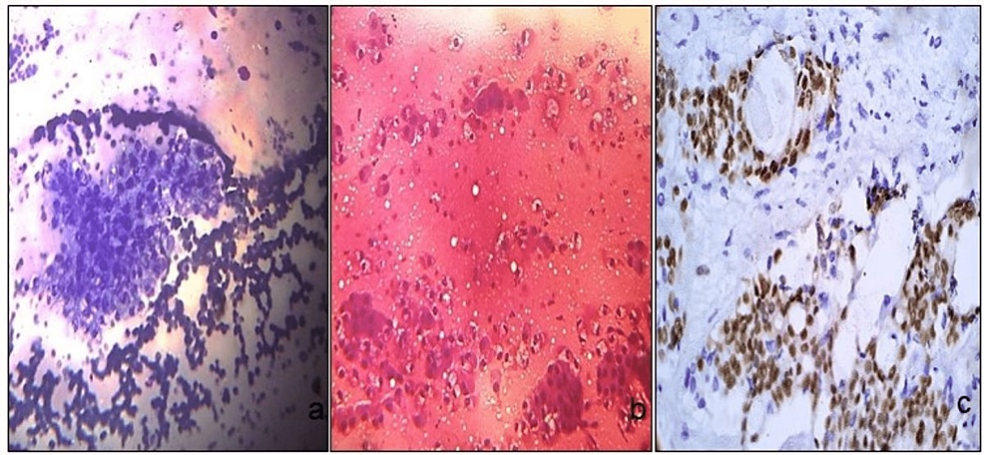
Stained cells. FNAC smears and cell block smears of mucoepidermoid carcinoma (a, b) (MGG stain, ×10 and H&E stain, ×20), and SOX-10 positive in tumor cells on cell block (c) (ICC stain, ×20). FNAC: fine-needle aspiration cytology, MGG: May-Grunwald-Giemsa, H&E: hematoxylin and eosin, ICC: immunocytochemistry.

**Figure 4:**
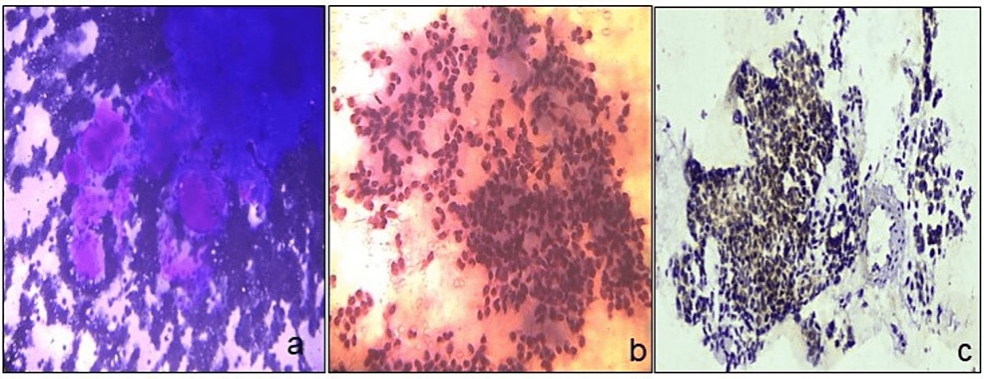
Stained cells. FNAC smears and Cell Block smear of Adenoid Cystic Carcinoma (a,b (MGG stain, ×10 and H&E Stain,×20),CD117 positive in tumor cells on cell block . (c)(IHC Stain, ×20)

**Figure 5:**
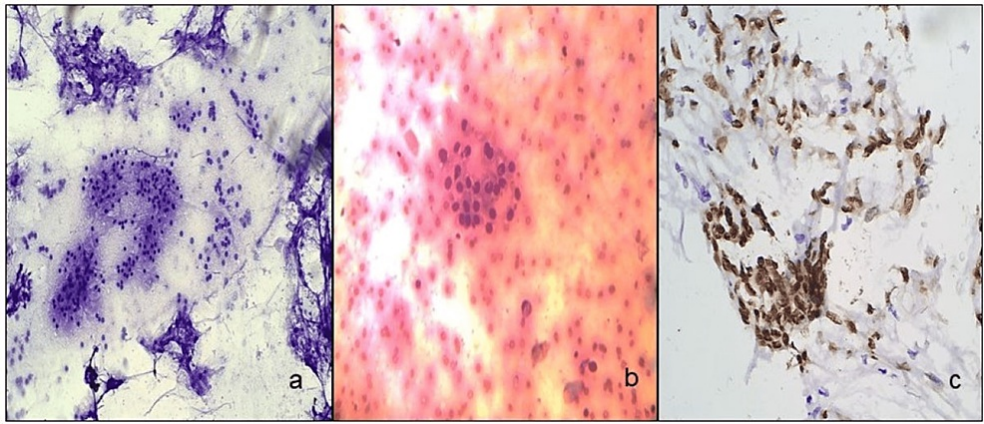
Stained cells. FNAC smears and cell block smears of acinic cell carcinoma (a, b) (MGG stain, ×10 and H&E stain,×20), and SOX-10 positive in tumor cells on cell block (c) (ICC stain, ×20). FNAC: fine-needle aspiration cytology, MGG: May-Grunwald-Giemsa, H&E: hematoxylin and eosin, ICC: immunocytochemistry.

Of the 60 cases enrolled in the study, classified according to the MSRSGC into six categories. Two cases (3.33%) were classified into Categories I and IV-b (non-diagnostic and salivary gland neoplasm of the salivary gland of uncertain malignancy), one case was classified into Category III (atypia of unknown significance), 28 cases (46.67%) were classified into Category IV-a (benign) (Figures 1, 2), five cases (8.33%) were in Category V (suspicious for malignancy), and the remaining 13 cases (21.67%) were in Category VI (malignant) (Figures 3-5) (Table 1).

**Table 1:** Distribution according to FNAC (Milan system) diagnosis (N=60). FNAC: fine-needle aspiration cytology.

FNAC diagnosis	No. of patients	Percentage
Non-diagnostic (Category 1)	2	3.33
Inadequate	2	3.33
Non-neoplastic (Category 2)	9	15.00
Benign cystic lesion	1	1.67
Acute sialadenitis	0	0
Chronic sialadenitis	7	11.67
Sialadenosis	1	1.67
Atypia of undetermined significance (AUS) (Category 3)	1	1.67
Neoplastic: benign (Category 4a)	28	46.67
Basal cell adenoma	2	3.33
Oncocytic neoplasm	1	1.66
Pleomorphic adenoma	22	36.6
Warthin's tumor	3	5.00
Neoplastic: Salivary gland neoplasm of uncertain malignancy (Category 4b)	2	3.33
Suspicious of malignancy (Category 5)	5	8.33
Malignant (Category 6)	13	21.67
Acinic cell carcinoma	2	3.33
Adenoid cystic carcinoma	5	8.33
Mucoepidermoid carcinoma (incl. low grade and intermediate)	6	10.00

FNAC diagnosis of patients in the Milan system was categorized into six different categories. A total of 60 cases were enrolled in the study, 12 cases (20.0%) were classified into Categories 1-3 (non-diagnostic, non-neoplastic, and atypia of unknown significance), 28 cases (46.67%) were benign (Category 4a), and the rest 20 cases (33.33%) were malignant (Categories 4a, 5, and 6). In cell block, five (8.33%) cases were not diagnostic, eight (13.33%) were non-neoplastic, 27 (45.00%) were diagnosed as benign, and the rest 20 (33.33%) were diagnosed as malignant (Table 2).

**Table 2:** Distribution of cases according to cell block diagnosis (N=60).

Cell block diagnosis	No. of patients	Percentage
Non-diagnostic	5	8.33
Inadequate	5	8.33
Non-neoplastic	8	13.33
Sialadenosis	1	1.67
Chronic sialadenitis	7	11.67
Benign	27	45.00
Basal cell adenoma	1	1.67
Pleomorphic adenoma	21	35.00
Warthin’s tumor	3	5.00
Oncocytic neoplasm	1	1.67
Sialolipoma	1	1.67
Salivary gland neoplasm of uncertain malignant potential	0	0.00
Suspicious of malignancy	1	1.67
Malignant	19	31.67
Acinic cell carcinoma	2	3.33
Adenoid cystic carcinoma	6	10.00
Epithelial myoepithelial carcinoma	1	1.67
Mucoepidermoid carcinoma	9	15.00
Salivary duct carcinoma	1	1.67

Histopathology was performed in only 47 cases, 24 (51.06%) were diagnosed as benign, and the rest 23 (48.93%) were diagnosed as malignant (Table 3). For the agreement of FNAC, cell block, and histopathology, only 45 cases were included for concordance of diagnosis, which were found to be significantly increased (Table 4). Inadequate or non-diagnostic, non-neoplastic in the diagnosis of FNAC, and cell block were excluded.

**Table 3:** Cytopathology and histopathology correlation (N=47). FNAC: fine-needle aspiration cytology, HPE: histopathological examination.

FNAC diagnosis (Milan Categories)	No. of cases on FNAC	HPE diagnosis	
		No. of cases on HPE	FNAC cases turned out to be benign or malignant on HPE
Non-diagnostic (Category 1)	2	0	0
Non-neoplastic (Category 2)	9	0	1: Benign
Benign cystic lesion	0	0	
Acute sialadenitis	0	0	
Chronic sialadenitis	7	0	
Sialadenosis	1	1	1: Sialolipoma
Atypia of undetermined significance (AUS) (Category 3)	1	1	1: Carcinoma ex pleomorphic adenoma
Neoplastic: benign (Category 4a)	28	25	19: Benign; 6: Malignant
Basal cell adenoma	2	0	1: Mucoepidermoid carcinoma; 1: Adenoid cystic carcinoma
Oncocytic neoplasm	1	0	1: Oncocytoma
Pleomorphic adenoma	22	0	15: Pleomorphic adenoma; 1: Acinic cell carcinoma; 2: Adenoid cystic carcinoma; 1: Epithelial myoepithelial carcinoma
Warthin's tumor	3	0	3: Warthin’s tumor
Neoplastic: Salivary gland neoplasm of uncertain malignancy (Category 4b)	2	2	I: Pleomorphic adenoma; 1: Carcinoma ex pleomorphic adenoma
Suspicious of malignancy (Category 5)	5	5	1: Pleomorphic adenoma; 2: Salivary duct carcinoma; 2: Mucoepidermoid carcinoma
Malignant (Category 6)	13	13	2: Benign; 11: Malignant
Acinic cell carcinoma	2	0	2: Acinic cell carcinoma
Adenoid cystic carcinoma	5	0	3: Adenoid cell carcinoma; 2: Pleomorphic adenoma
Mucoepidermoid carcinoma	6	0	6: Mucoepidermoid cell carcinoma

**Table 4:** Agreement of FNAC and cell block diagnosis, cell block and histopathology (HPE), and FNAC and histopathology (N=45). FNAC: fine-needle aspiration cytology, PPV: positive predictive value, NPV: negative predictive value, HPE: histopathological examination.

Diagnosis	Sensitivity	Specificity	PPV	NPV	Diagnostic accuracy
FNAC and cell block	80.0	84.0	80.0	84.0	82.2
FNAC and HPE	72.7	82.6	80.0	76.0	77.8
Cell block and HPE	82.6	95.5	95.0	84.0	88.9

The agreement of the FNAC Milan system and cell block diagnosis was observed for 37/45 (82.2%) to detect benign and malignant salivary gland lesions. The diagnostic efficacy of the FNAC Milan system compared to the cell block diagnosis in terms of sensitivity, specificity, PPV, and NPV was 80.0%, 84.0%, 80.0%, and 84.0%, respectively. The diagnostic precision of the FNAC Milan system was 82.2% as compared to the cell block (k=0.640; p<0.001).

The agreement of cell block and histopathological diagnosis was observed for 40/45 (88.9%). The diagnostic efficacy of the cell block diagnosis (as compared to histopathology) in terms of sensitivity, specificity, PPV, and NPV was 82.6%, 95.5%, 95.0%, and 84.0%, respectively. The diagnostic precision of cell block was 88.9% compared to histopathology (k=0.778; p<0.001).

The agreement of the FNAC Milan system and histopathology was observed for 35/45 (77.8%). The diagnostic efficacy of the FNAC Milan system (compared to histopathology) in terms of sensitivity, specificity, PPV, and NPV was 72.7%, 82.6%, 80.0%, and 76.0%, respectively. The diagnostic precision of the FNAC Milan system was 77.8% compared to histopathology (k=0.554; p<0.001).

Of 60 FNAC cases, histopathology was available in only 47 cases, 24 (51.07%) were diagnosed as benign, and the rest 23 (48.93%) were diagnosed as malignant neoplasm. Histopathology of Category I was not available. In Category II, out of nine cases, only one case of histopathology was available which turned out to be benign. In Category III, one case turned out to be malignant in histopathology. In Category IVa, we have 28 cases in FNAC; in histopathology, only 25 cases were available, 19 cases were benign, and six cases were found to be malignant. In Category IVb, only two cases were there; among these, one case turned out to be benign and one case was malignant. In five cases of Category V, one case turned out to be benign and the rest of the four cases were malignant. In Category VI, out of 13 cases, two cases turned out to be benign, and the remaining 11 cases turned out to be malignant (Table 3). In the present study, for FNAC Milan Categories I, II, III, IVa, IVb, V, and VI, the risk of malignancy is, respectively, 0%, 0%, 100%, 24%, 50%, 80%, and 84.6% (Tables 5, 6).

**Table 5:** Histological follow-up of FNAC according to Milan system diagnostic categories. FNAC: fine-needle aspiration cytology, Cat.: Category.

	Cat. I	Cat. II	Cat. III	Cat. IVa	Cat. IVb	Cat V	Cat VI	Total
No. of cases	2 (3.3%)	9 (15.0%)	1 (1.7%)	28 (46.7%)	2 (3.3%)	5 (8.3%)	13 (21.7%)	60
No. of histological follow-up	0	1	1	25	2	5	13	47
Benign	0	1	0	19	1	1	2	24
Malignant	0	0	1	6	1	4	11	23
Risk of malignancy	0.0	0.0	100.0	24.0	50.0	80.0	84.6	48.9

**Table 6:** Malignancy risk for different Milan categories as reported in different contemporary studies and their comparison with the present study.

Milan Category	Malignancy risk %				
	Amita et al. [13]	Rohilla et al. [14]	Karuna et al. [15]	Bharti et al. [16]	Present study
I (Inadequate)	0	0	0	0	0
II (Non-neoplastic)	6.3	17.4	0	0	0
III (Atypia of undetermined significance)	100	100	50	100	100
IVa (Neoplastic benign)	0	7.3	2.44	5.3	24
IVb (Neoplastic uncertain malignant potential)	25	50	33.3	33.0	50
V (Suspicious)	100	0	100	0	80
VI (Malignant)	100	96	93.3	80	84.6

Immunocytochemical markers were applied on the cell block section for exact tumor subtyping. In benign cases like pleomorphic adenoma, we used ICC markers such as P63, CK7, and SOX-10 and reported the nuclear, cytoplasmic, and nuclear positivity of markers. However, Warthin's tumor was tested for leukocyte common antigen (LCA) and CK7. On the contrary, in malignant cases like carcinoma ex pleomorphic adenoma markers Plag-1, CK7, and P63 were used. Similarly, mucoepidermoid carcinoma was tested for S-100, SOX-10, and P63; acinic cell carcinoma was tested for SOX-10 and CK7; and adenoid cystic carcinoma was tested for markers, CD117, SOX-10, and Calponin, respectively.

## Discussion

Salivary gland tumors are known to have a heterogeneous profile with variable clinical presentation, histopathology, and malignancy rate. The diagnosis of salivary gland lesions is difficult and challenging [4]. They comprise nearly 2%-6.5% of tumors in the head and neck region and are of mainly epidermal origin [5-7]. In recent years, fine-needle aspiration cytology has emerged as a useful, effective, reliable, and popular method for the evaluation of salivary gland lesions [8-11]. Despite the popularity of FNAC in the diagnosis of salivary gland tumors, reporting of cytological results was not standardized. Recently, a global consensus was developed regarding reporting of FNAC results to categorize salivary gland lesions in accordance with their malignancy potential [12]. Subsequently, its applicability in terms of stratification of the risk of malignancy of salivary gland tumors has been evaluated in different parts of the world. In the present study, we also attempted to assess the applicability of the Milan system for reporting salivary gland cytology along with the usefulness of a panel of ICC markers for the diagnosis of salivary gland neoplasms at our center. In this prospective study, 60 patients were enrolled for salivary gland lesions who underwent FNAC, cell block, and ICC and were correlated with histopathology.

The ages of the patients ranged from 15 to 75 years. The majority of them were aged 19 to 45 years (51.7%), 35% were female and the rest were males (65%). In a recent study from Karnataka, Amita et al. [13] also reported a wide age range of patients with ages ranging from nine years to 83 years (mean age: 48.3 years) and a dominance of men (67.7%). Rohilla et al. [14] in another study from Chandigarh reported that the age range of the patients ranged from one to 95 years (mean age: 43.7 years) and the proportion of men was 63.0%. Karuna et al. [15] in another study from north India (Meerut) found 105 patients aged less than 10 years to more than 61 years of age. The dominance of patients between 31 and 60 years was 54.3% and the total men percentage was 69.5%. Bharti et al. [16] in a study from Rajasthan reported the age range at three to 86 years (mean age: 39 years) and the proportion of men at 61.3%. Therefore, the age and sex profile of the patients in the present study are comparable to that of different contemporary reports from various parts of India. In the present study, most of the cases (96.7%) had unilateral involvement. Only two (3.3%) cases had bilateral involvement. The right side was more commonly involved (32/62; 51.6%) compared to the left side (30/62; 48.4%). Among the different sites, the parotid gland (65%) was the most frequently involved followed by the submandibular region (23.3%). Compared to the present study, Rohilla et al. [14] in their study reported the dominance of the parotid gland (61.3%) and the submandibular gland (35.7%). In the study by Karuna et al. [15], the proportion of those with involvement of the parotid and submandibular glands was 62.4% and 31.2%, respectively.

In the present study, among benign non-neoplastic lesions (n=28), the most common diagnosis was pleomorphic adenoma (n=22) followed by Warthin’s tumor (n = 3), respectively. Amita et al. [13] in their study reported 68 non-neoplastic lesions; however, they found pleomorphic adenoma (n=22) and Warthin's tumor (n=6) as benign subtypes. Similar observations were also made by Karuna et al. who [15] identified 41/54 and 6/54 of their benign neoplastic lesions as pleomorphic adenoma and Warthin's tumor, respectively. Bharti et al. [16] in their study identified 66/85 and 10/85 benign non-neoplastic lesions such as pleomorphic adenoma and Warthin's tumor, respectively. The findings of the present study thus do not show a difference in the cytological diagnosis of non-neoplastic benign lesions from the other contemporary Indian studies.

In the present study, among the 13 malignant lesions, the maximum (n=6) was diagnosed as mucoepidermoid carcinoma followed by adenoid cystic carcinoma (n=5) and acinic cell carcinoma (n=2), respectively. In the study by Amita et al. [13] among 23 malignant lesions, 12 were diagnosed as mucoepidermoid carcinoma, four as acinic cell carcinoma, and three as squamous cell carcinoma while a total of four (one each) comprised other malignant conditions. However, Rohilla et al. [14] who identified 61 malignant cases identified maximum cases (n=20) as carcinoma not otherwise specified (NOS) followed by mucoepidermoid carcinoma (n=15) and adenoid cystic carcinoma (n=8) as the main diagnoses. Karuna et al. [15] in their series of 17 malignant FNAC also diagnosed mucoepidermoid carcinoma (n=9) as the most common malignant type followed by adenoid cystic carcinoma (n=3) as the next most common malignant type. In the study by Bharti et al. [16] as many as eight malignant types were diagnosed in a total of 35 malignant cases with a total of 14 diagnosed as mucoepidermoid carcinoma as the most dominant type. The correlation of the findings of the present study with other studies also shows the dominance of mucoepidermoid carcinoma as the most common malignancy (Table 3). The usefulness of fine-needle aspiration (FNA) as a preoperative diagnostic procedure was studied in 43 patients with salivary gland tumors. Nine of the tumors were malignant and 34 benign. The diagnostic sensitivity of FNA was 88.9% (8/9), the specificity 94.1% (32/34), and the accuracy 93.0% (40/43). These results indicate that FNA is a highly sensitive and specific screening procedure [17].

In the present study, the non-neoplastic category (Category 2) was the other major diagnostic category according to the Milan system (n=9). In this category, the maximum was diagnosed as chronic sialadenitis (n=7). There was one case each confirmed as benign cystic lesion and sialadenosis, respectively. Although, Amita et al. [13] too found chronic sialadenitis as the main type of non-neoplastic lesion (n=37/68); however, in their study, acute sialadenitis (n=26/68) also comprised a substantial proportion of non-neoplastic cases. However, Rohilla et al. [14] in their study categorized 352 cases as non-neoplastic, but benign neoplastic lesions were found in 188 patients and malignant lesions were found in 61 patients. In their study, sialadenitis was diagnosed as a single category (chronic/acute) and was the next most common non-neoplastic type (n=141). In the study by Karuna et al. [15] of the 17 non-neoplastic cases, seven were chronic sialadenitis and three were acute sialadenitis, while a total of six were diagnosed as sialadenosis. Bharti et al. [16] on the other hand in a series of 42 non-neoplastic category lesions found sialadenitis as the most common (n=16); however, the next most common type was a benign cyst (n=12) in their study. In fact, although there might be some differences in the spectrum of non-neoplastic lesions, the findings of the present study in accordance with other contemporary studies from India also show the dominance of sialadenitis as the most common non-neoplastic type (Table 6).

In the present study, we also carried out cell block and immunocytochemical evaluation. On evaluation of cell block, five (8.33%) cell block sections were not diagnostic, eight (13.3%) were non-neoplastic, 27 (45%) were benign, one (1.7%) was neoplasm of uncertain potential for malignancy, one (1.7%) was suspected of malignancy, and 19 (31.7%) were malignant according to the reporting category of the Milan system. Although FNAC is considered to be a quite popular primary cytological assessment for salivary gland tumors, it is often limited for some specific diagnoses such as pleomorphic adenoma and Warthin's tumor. This is all because there can be problems with poor cellularity or aspirate quality. In order to overcome these issues, we use additional modalities, such as the preparation of cell blocks and immunocytochemistry. Cell block enables evaluating the histological pattern of the disease that is otherwise not possible in the case of FNAC smears.

The purpose of using cell block with ICC and FNAC was to improve the final diagnosis. Using cell block helped to identify 18 malignancies instead of 13 cases of FNAC. In total, FNAC diagnosed 18 cases as suspected of malignancy/malignancy. When cell block was used, a total of 20 cases were diagnosed as suspected of malignancy and malignancy. Thus, cell block not only helped to increase the overall detection of malignancies but also helped to achieve conclusive malignant diagnosis in a substantial number of cases.

In a previous study, Naz et al. [18] too found that cell block with immunocytochemistry helps to improve the diagnosis of salivary gland lesions. In the final assessment against the histopathological assessment, we also found that cell block helped to improve both sensitivity and specificity (82.6% and 95.5%) compared to FNAC (80% and 84%), respectively. Similarly, to the findings of the present study, Oberoi et al. [19] also reported an increment in sensitivity from 77.8% for FNAC to 88.8% for cell block. Wadhwa et al. [20] too in an evaluation of head and neck lesions found an increase in both sensitivity and specificity from 88.8% and 95.7% by FNAC to 96% and 100%, respectively, for cell block with ICC. The findings of the present study are also in agreement with this.

In the present study, for the Milan Categories I, II, III, IVa, IVb, V, and VI, the risk of malignancy was 0%, 0%, 100%, 24%, 50%, 80%, and 84.6% respectively. A comparison of the findings of the present study with contemporary studies shows a minimal risk of malignancy in Categories I, II, IVa, and IVb, while the maximum risk is in Categories III, V, and VI. These findings show that the Milan system plays a useful role in risk stratification. There are few studies that indicate a lesser number of cases in Categories V to VI, among which, all are having maximum risk of malignancy [14,16]. However, we suggest the use of cell block in such a situation as it helps to greatly improve the diagnosis of Category V lesions. One of the limitations of the present study was the small sample size, due to which the proportional (%) value of some of the categories in risk stratification became too high. Further studies are recommended with a larger sample size. Given the improvisation value of cell block and the ability to report its findings in the Milan system, it is recommended to use cell block as a complementary tool to FNAC along with risk stratification using the Milan system of reporting. Furthermore, increasing the availability of ICC markers for salivary gland tumors will enhance the possibility that salivary gland FNAC plays an increasingly essential role in the evaluation of salivary gland lesions.

Limitations of the study

Our study had various limitations; sample size was comparatively small. A large sample size and multicentric study with high precision and accuracy must be recommended for a more reliable interpretation of results. Furthermore, due to COVID-19, the limited flow of patients to our tertiary care center made it difficult to design our study in a larger group.

## Conclusions

Some important factors that can help improve the diagnostic accuracy of FNAC of salivary gland lesions include cell block and ancillary techniques such as immunocytochemistry. We recommend a multi-centric investigation with high descriptive sample size. Most importantly, we stratified all salivary gland lesions according to MSRSGC and thus tried to evaluate its diagnostic accuracy and calculate the risk of malignancy for each category and provide useful information for the clinical management of the patient.
